# Usefulness of Urea as a Means of Improving the Solubility of Poorly Water-Soluble Ascorbyl Palmitate

**DOI:** 10.1155/2017/4391078

**Published:** 2017-11-06

**Authors:** Yutaka Inoue, Daichi Niiyama, Isamu Murata, Ikuo Kanamoto

**Affiliations:** Laboratory of Drug Safety Management, Faculty of Pharmacy and Pharmaceutical Sciences, Josai University, 1-1 Keyakidai, Sakado-shi, Saitama 3500295, Japan

## Abstract

The aim of this study was to evaluate complexes of L-ascorbyl palmitate (ASCP) and urea (UR). This evaluation involved differential scanning calorimetry (DSC), powder X-ray diffraction (PXRD), scanning electron microscopy (SEM), near-infrared spectroscopy (NIR), a solubility test, a 1,1-diphenyl-2-picrylhydrazyl (DPPH) radical scavenging test, and a mushroom tyrosinase inhibition assay. Physicochemical evaluation revealed that ASCP/UR complexes form at a molar ratio of 1/12. The solubility test revealed that ASCP/UR complexes had increased solubility compared to ASCP. The DPPH radical scavenging test and mushroom tyrosinase inhibition assay revealed that the activity of ASCP/UR complexes was not impaired by complex formation. These results are probably due to the tetragonal crystal system of UR changing to a hexagonal crystal system and interaction with the alkyl group of ASCP.

## 1. Introduction

Ascorbic acid (AA) has antioxidant effects and is widely used as a main ingredient in cosmetics to prevent spots and wrinkles by scavenging reactive oxygen species (ROS) [1, 2]. ROS play a major role in damage due to ultraviolet irradiation. ROS stimulate and activate melanocytes in the basal layer of epidermis, prompting melanocytes to produce the black pigment melanin. Melanin normally acts to protect the skin from ultraviolet light, and melanin is removed as the skin turns over. If, however, the skin is overexposed to ultraviolet light for a prolonged period, then spots are produced. In addition, ROS oxidize and degrade collagen, a key component of the dermis. Degradation of collagen damages the elasticity of the skin, so ROS cause wrinkles. In addition, AA is known to promote collagen production and to inhibit the generation of melanin [3–5]. However, AA is unstable when exposed to heat, light, oxygen, or acidic media, and AA readily oxidizes in aqueous solution [6, 7]. Thus, various derivatives have been synthesized to improve the stability of AA [8]. One such derivative is ascorbyl palmitate (ASCP), which is an ester formed from AA and palmitic acid. ASCP is stable when exposed to heat and light, and it retains AA's antioxidant effects and its action to inhibit the generation of melanin. ASCP is highly fat-soluble since it includes palmitic acid. A lipophilic drug has exceptional skin penetration but poor solubility. This presents a dilemma since a product cannot contain a sufficient quantity of a lipophilic drug. Thus, improving the solubility of ASCP will help develop products containing ASCP and facilitate the expanded use of ASCP. In general, it was known that as a means of improving solubility self-micellization with AA and a poorly water-soluble drug, cocrystallization with nicotinamide and urea (UR) is carried out [9, 10].

UR has a moisturizing effect by hydrating the stratum corneum and loosening and removing keratin. A moisturizer and percutaneous penetration enhancer, UR is used as a main ingredient in or additive to pharmaceuticals and cosmetics [11]. In addition, UR is hydrotropic, and this organic compound dissolves in water at high concentrations [12]. Hydrotropes are used to increase the solubility of drugs such as diclofenac. UR normally has a tetragonal crystal system but as a polymorph it has a hexagonal crystal system [13, 14]. UR with a hexagonal crystal system differs from UR with a tetragonal crystal system, and UR with a hexagonal crystal system is known to include small molecules in its voids and it has increased solubility [15]. Studies have mentioned palmitic acid, indomethacin, and clarithromycin as drugs that form complexes [16, 17]. Thus, ASCP containing palmitic acid may form complexes with UR with a hexagonal crystal system and potentially exhibit improved solubility.

Accordingly, the current study used UR as a solubilizing agent to prepare ASCP/UR complexes and to assess the properties, solubility, antioxidant effects, and action to inhibit the generation of melanin of those complexes in order to improve the solubility of ASCP.

## 2. Materials and Methods

### 2.1. Materials

**Figure 1:**
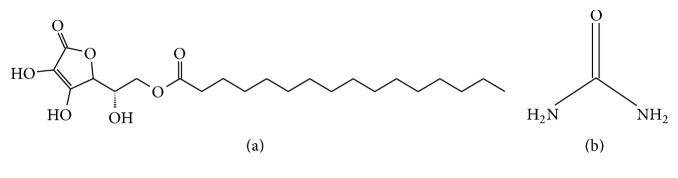
Chemical structure. (a) ASCP; (b) UR.

ASCP from Tokyo Chemical Industry was used in this study (Figure 1(a)). UR from Wako Pure Chemical Industries was also used (Figure 1(b)). All other reagents were from Wako Pure Chemical Industries and were of special reagent grade.

### 2.2. Preparation of a Physical Mixture (PM) and a Concentrated Sample

A physical mixture (PM) was prepared by weighing ASCP and UR in a certain molar ratio (1/6 or 1/12) and then mixing the two with a Vortex Mixer for 1 min. A concentrated sample was prepared by solvent evaporation (EVP). A PM (200 mg) was dissolved in 20 mL of methanol, the solvent was evaporated at 40°C, and the concentrated sample was centrifuged at 25 rpm to form crystals.

### 2.3. Differential Scanning Calorimetry (DSC)

A high-sensitivity differential scanning calorimeter (Thermo Plus Evo, Rigaku) was used to perform DSC. About 2 mg of a measured sample was placed in a sealed aluminum pan, and DSC was performed in a nitrogen gas atmosphere (60 mL/min) with temperature increasing at a rate of 5°C/min.

### 2.4. Powder X-Ray Diffraction (PXRD)

A Mini Flex II powder X-ray diffractometer (Rigaku) was used to perform PXRD. PXRD was performed using Ni-filtered CuK*α* (30 kV, 15 mA) as an X-ray source. Conditions for PXRD were a scan rate of 4°/min over a scan range of 2*θ* = 5–35°. Each powdered sample was evenly spread on a glass plate and PXRD was then performed.

### 2.5. Scanning Electron Microscopy (SEM)

The S3000 N Scanning Electron Microscope (Hitachi High-Technologies) was used to perform SEM. Gold was deposited on each sample for 70 s, and microscopy was performed at an accelerating voltage of 10 kV.

### 2.6. Near-Infrared (NIR) Spectroscopy

The Buchi NIR Flex N-500 spectrometer (Nihon Buchi) was used to perform NIR spectroscopy. Each sample was placed in a sample cup, and spectra were collected for 8 s in the range of 10000–4000 cm^−1^ at 25°C using a cell with a 1 mm optical path length.

### 2.7. Solubility Test

ASCP was dispersed in distilled water to reach a concentration of 0.5 mg/mL, and the mixture was shaken for 0, 1, 3, 6, 12, and 24 h at 37°C. Afterwards, the mixture was filtered with a 0.45 *μ*m cellulose acetate membrane filter. The resulting solid served as the sample, and the sample was assayed using HPLC. The assay was performed using the Waters e2795 ultraviolet-visible spectrophotometer (Nihon Waters) at a wavelength of 266 nm. The HPLC column used was the Cosmosil 5C_18_-AR-II Packed Column (*φ*5 *μ*m, 4.6 mm ID × 250 mm), the sample injection volume was 100 *μ*L, and the column temperature was 40°C. A methanol/acetic acid (pH 6.5) mixture (85/15) was used as the mobile phase, and the retention time for ASCP was set at about 9 min.

### 2.8. DPPH Radical Scavenging Test

The Spectra Max 190 (Molecular Devices Japan) was used to perform a DPPH radical scavenging test. A DPPH methanol solution (100 *μ*M) and a methanol solution of each sample were mixed in a microplate at a volume ratio of 1/1, the mixture was incubated for 5 min at 25°C, and then the absorbance of DPPH was read at a wavelength of 517 nm. A mixture of a DPPH methanol solution/methanol (1/1) served as a negative control (*A*_0_), and methanol served as a positive control (Br). The percent DPPH radical scavenging activity was calculated using

(1) Radical  scavenging  activity=1−As−BrA0−Br∗100.

### 2.9. Mushroom Tyrosinase Inhibition Assay

The Spectra Max 190 (Molecular Devices Japan) was used to perform a DPPH radical scavenging test. Fifty *μ*L of L-tyrosine (2 mM), 90 *μ*L of 0.1 M phosphate buffer (pH 6.8), and 10 *μ*L of DMSO with or without a sample were added to a 96-well microplate. Fifty *μ*L of phosphate buffer with or without mushroom tyrosinase (200 units) was added, before the mixture was incubated for 20 min at 37°C, and the amount of DOPA-chrome was determined at 405 nm. DMSO/phosphate buffer (1/9) served as a negative control (*A*_0_), and phosphate buffer served as a positive control (Br). The percent mushroom tyrosinase inhibition activity was calculated using

(2) Mushroom  inhibitory  activity=1−As−BrA0−Br∗100.

## 3. Results and Discussion

### 3.1. DSC

When UR with a hexagonal crystal system included small molecules in its voids, the melting point of UR drops according to one study [13]. Another study has reported that stability is improved by UR complex formation and the amount of UR that melts increases [17]. Therefore, the current study used DSC to determine the molar ratio and thermal properties of ASCP/UR complexes.

**Figure 2:**
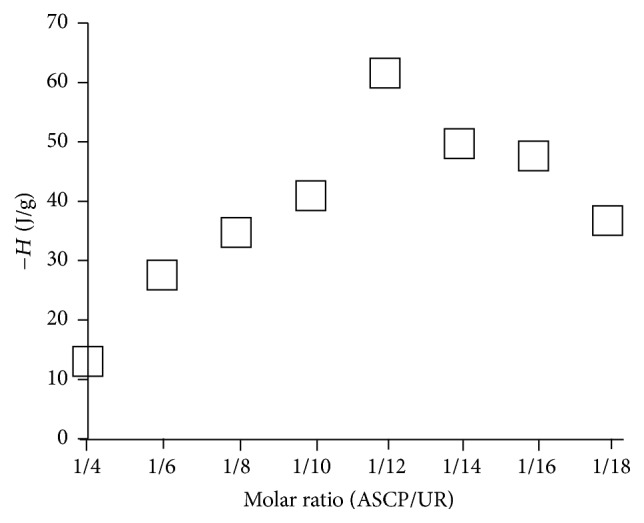
Heat of fusion of ASCP/UR systems.

The molar ratio of ASCP/UR complexes was determined based on the difference in thermal stability (Figure 2). ASCP/UR at a molar ratio of 1/12 had the highest heat of fusion and the highest thermal stability. Thermal stability is known to improve as a result of complex formation with UR. The optimum molar ratio of ASCP/UR is presumably 1/12 because ASCP/UR is most stable against heat at a molar ratio of 1/12. Moreover, ASCP/UR at a molar ratio of 1/6 had a lower heat of fusion because of the excessive amount of ASCP.

**Figure 3:**
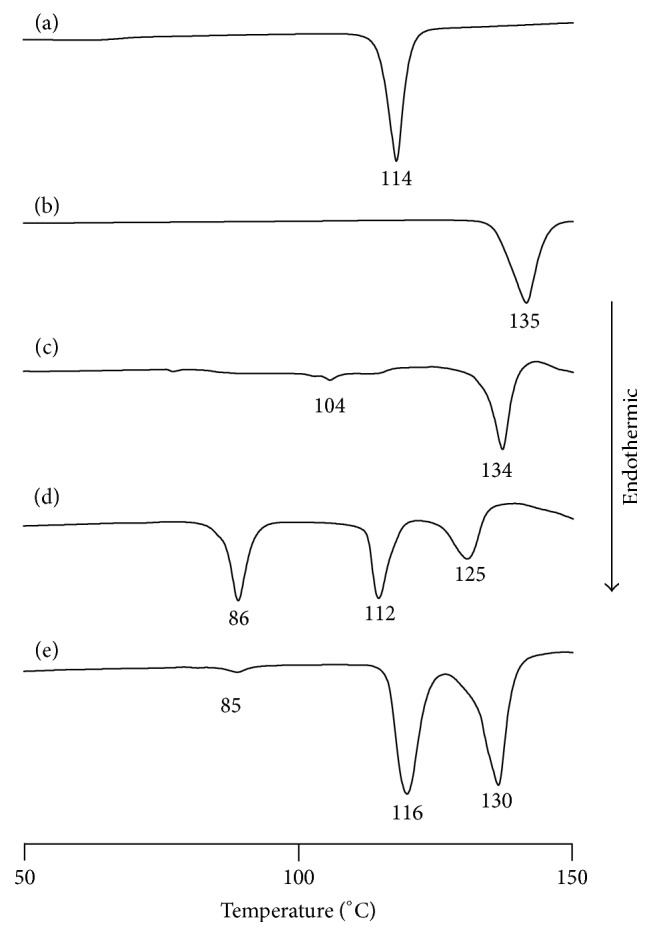
DSC curves of ASCP/UR systems. (a) ASCP, (b) UR, (c) PM (ASCP/UR at a molar ratio of 1/12), (d) EVP1 (ASCP/UR at a molar ratio of 1/6), and (e) EVP2 (ASCP/UR at a molar ratio of 1/12).

DSC was performed to examine the thermal behavior of ASCP/UR complexes (Figure 3). UR and the PM (ASCP/UR at a molar ratio of 1/12) had an endothermic peak due to UR at 134°C. The endothermic peak due to UR shifted to 125°C and 130°C at lower temperatures for EVP1 (ASCP/UR at a molar ratio of 1/6) and EVP2 (ASCP/UR at a molar ratio of 1/12). When UR forms complexes, the endothermic peak is known to decrease. With EVP1 (ASCP/UR at a molar ratio of 1/6) and EVP2 (ASCP/UR at a molar ratio of 1/12), the endothermic peak due to UR was lower. This suggested complex formation in EVP1 (ASCP/UR at a molar ratio of 1/6) and EVP2 (ASCP/UR at a molar ratio of 1/12). With EVP1 (ASCP/UR at a molar ratio of 1/6) and EVP2 (ASCP/UR at a molar ratio of 1/12), an endothermic peak was observed at 85°C. An endothermic peak is associated with a change from UR with a tetragonal crystal system to UR with a hexagonal crystal system at a lower temperature than the endothermic peak produced by UR alone. Therefore, the endothermic peak observed at 85°C with EVP1 (ASCP/UR at a molar ratio of 1/6) and EVP2 (ASCP/UR at a molar ratio of 1/12) was an endothermic peak associated with a change from UR with a tetragonal crystal system to UR with a hexagonal crystal system. This suggests that UR has a hexagonal crystal system in EVP1 (ASCP/UR at a molar ratio of 1/6) and EVP2 (ASCP/UR at a molar ratio of 1/12).

### 3.2. PXRD

**Figure 4:**
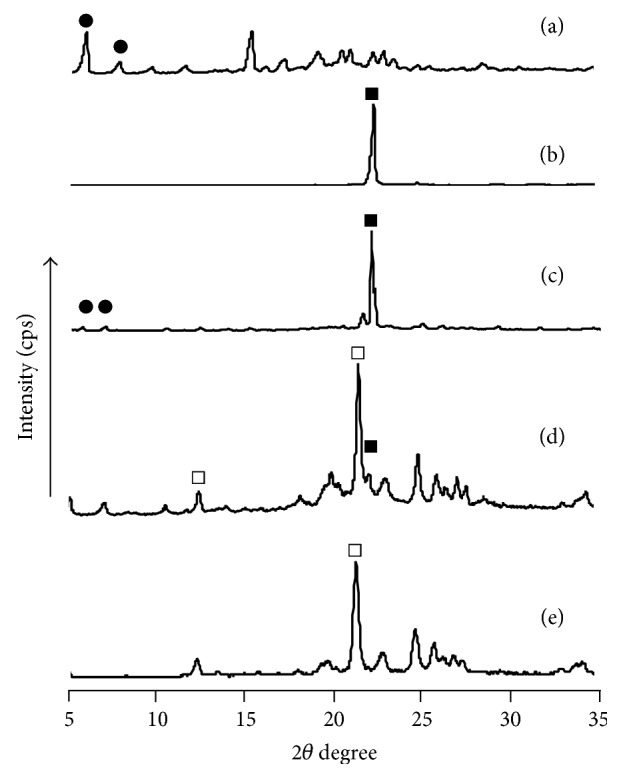
PXRD patterns of ASCP/UR systems. (a) ASCP, (b) UR, (c) PM (ASCP/UR at a molar ratio of 1/12), (d) EVP1 (ASCP/UR at a molar ratio of 1/6), and (e) EVP2 (ASCP/UR at a molar ratio of 1/12). ●: specific peak of ASCP; □: specific peak of UR with a tetragonal crystal system; ■: specific peak of UR with a hexagonal crystal system.

DSC results suggested complex formation in EVP2 (ASCP/UR at a molar ratio of 1/12) and that the tetragonal crystal system of UR changed into a hexagonal crystal system as a result of complex formation. The characteristic diffraction pattern of UR with a hexagonal crystal system is reported to be 2*θ* = 22.0° and 24.4°. In contrast, the characteristic diffraction pattern of UR with a tetragonal crystal system is 2*θ* = 12.3° and 21.5° [13]. Accordingly, the crystalline state of ASCP/UR was evaluated with PXRD and complex formation was examined (Figure 4).

With ASCP and the PM (ASCP/UR at a molar ratio of 1/12), characteristic peaks due to ASCP (●) were observed at 2*θ* = 7.6° and 9.5°. In addition, with UR and PM (ASCP/UR at a molar ratio of 1/12), a characteristic peak due to UR (■) was observed at 2*θ* = 22.0°. With EVP2 (ASCP/UR at a molar ratio of 1/12), there were no peaks characteristic of ASCP (●) and UR (■). A new peak due to UR (□) was observed at 2*θ* = 21.5°. This peak coincides with the characteristic peak of UR with a hexagonal crystal system. Therefore, UR in EVP2 (ASCP/UR at a molar ratio of 1/12) conceivably has a hexagonal crystal system, which is why ASCP/UR complexes are formed.

### 3.3. SEM

**Figure 5:**
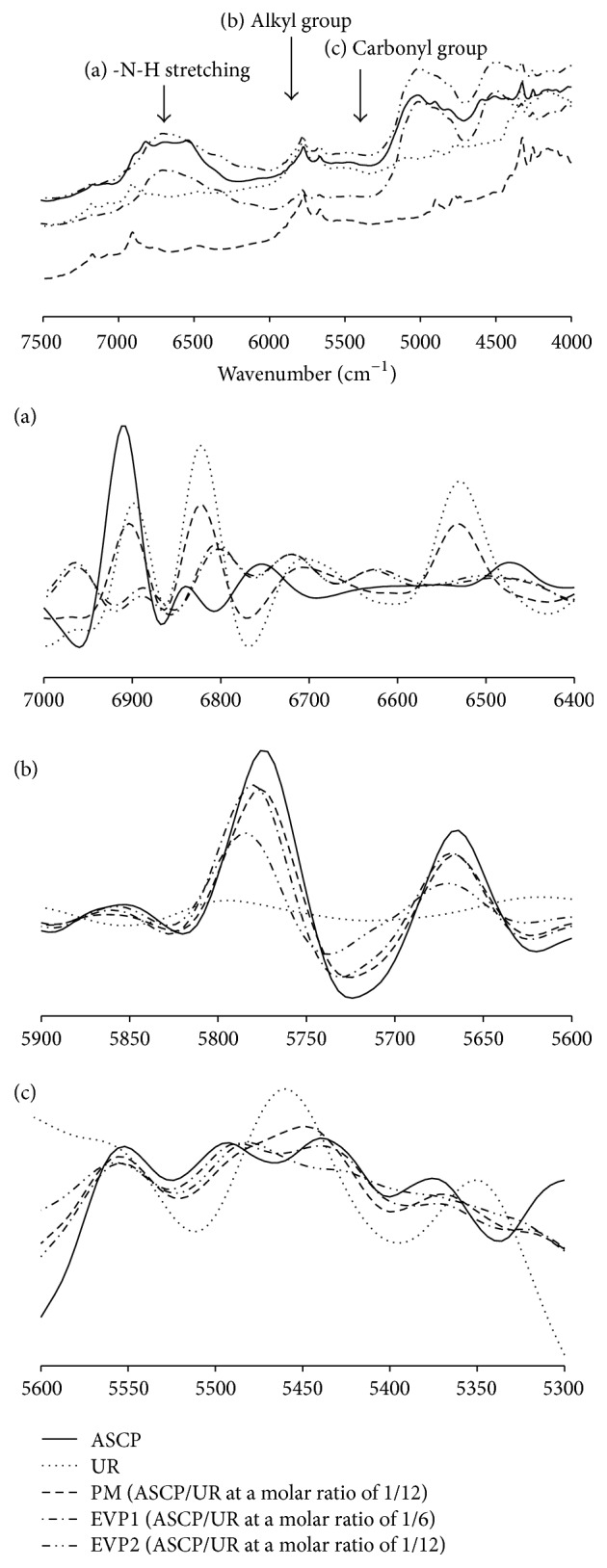
NIR absorption spectra of ASCP/UR systems and second-differentiation NIR absorption spectra of ASCP/UR systems ((a) -N-H stretching, (b) alkyl group, and (c) carbonyl group).

Results of DSC and PXRD suggested complex formation in EVP2 (ASCP/UR at a molar ratio of 1/12). In addition, PXRD results indicated that the crystal state of UR changes. UR is reported to have crystals with a hexagonal system when forming a complex with a drug. For example, Debbarma et al. reported that “urea as an organic base produced the micro-sized hexagonal shape structure with zinc sulphate” [18]. Therefore, the current study performed SEM to observe the shape and surface of crystals (Figure 5). The surface of ASCP was rough and acicular crystals were aggregated. The surface of UR was smooth and had columnar crystals. In EVP2 (ASCP/UR at a molar ratio of 1/12), smooth and glassy crystals were observed. In addition, crystals with what appeared to be a hexagonal system were observed. SEM revealed crystals with a hexagonal system in EVP2 (ASCP/UR at a molar ratio of 1/12), suggesting ASCP/UR complex formation.

### 3.4. NIR

**Figure 6:**
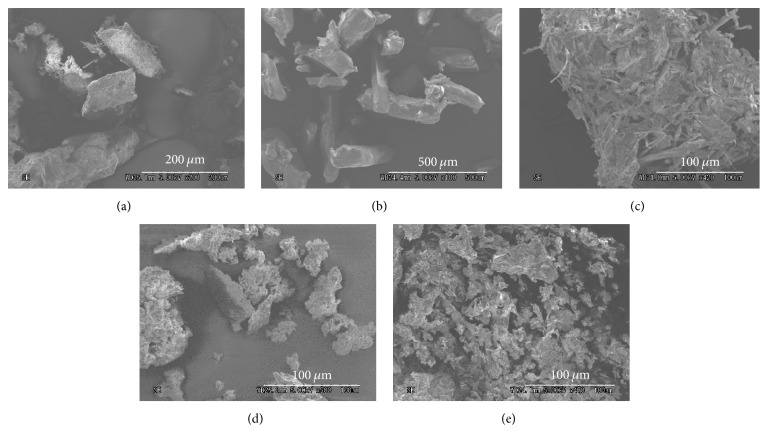
SEM morphology of ASCP/UR systems. (a) ASCP, (b) UR, (c) PM (ASCP/UR at a molar ratio of 1/12), (d) EVP (ASCP/UR at a molar ratio of 1/6), and (e) EVP (ASCP/UR at a molar ratio of 1/12).

DSC, PXRD, and SEM results suggested complex formation in EVP2 (ASCP/UR at a molar ratio of 1/12). Therefore, NIR was performed in order to investigate more detailed intermolecular interaction between ASCP and UR (Figure 6). A study has reported that the change in the crystal state of UR accompanying the transition from a tetragonal crystal system to a hexagonal crystal system results in a change in the C-O bond distance from 1.25 Ǻ to 1.28 Ǻ and a change in the H-O bond distance from 2.99 Ǻ to 2.93 Ǻ [15]. ASCP and the PM (ASCP/UR at a molar ratio of 1/12) had peaks due to the alkyl group (-CH_2_-) of ASCP at 5664 cm^−1^ and 5772 cm^−1^ and a peak due to the carbonyl group (-C=O-) of ASCP at 5444 cm^−1^ while UR had peaks due to in-phase vibration of N-H of UR at 6820 cm^−1^ and out-phase vibration of N-H of UR at 6532 cm^−1^. With EVP2 (ASCP/UR at a molar ratio of 1/12), the peak for the alkyl group of ASCP shifted to a higher wavenumber (5788 cm^−1^) than the peak for that group in ASCP. In addition, the peak for the in-phase vibration of N-H in EVP2 (ASCP/UR at a molar ratio of 1/12) shifted to a lower wavenumber (6786 cm^−1^) than the peak for that group in the UR. Similarly, the peak for the out-phase vibration of N-H in EVP2 (ASCP/UR at a molar ratio of 1/12) shifted to a higher wavenumber (6624 cm^−1^) than the peak for that group in UR. DSC, PXRD, and SEM results suggested that UR has a hexagonal crystal system and that it interacts with ASCP. With EVP2 (ASCP/UR at a molar ratio of 1/12), the peak due to stretching vibration of the alkyl group of ASCP shifted to a higher wavenumber, the peak due to antisymmetric stretching vibration of N-H of UR shifted to a lower wavenumber, and the peak due to symmetrical stretching vibration of N-H shifted to a higher wavenumber. These characteristics were presumably due to the change in the intermolecular distance as a result of complex formation. The peak due to stretching vibration of the carbonyl group of ASCP shifted to a lower wavenumber in EVP2 (ASCP/UR at a molar ratio of 1/12). However, no shift was observed in EVP1 (ASCP/UR at a molar ratio of 1/6). This suggests that EVP1 (ASCP/UR at a molar ratio of 1/6) forms complexes with ASCP/UR at a molar ratio of 1/12 and that an excessive amount of ASCP was present.

### 3.5. Solubility Test

**Figure 7:**
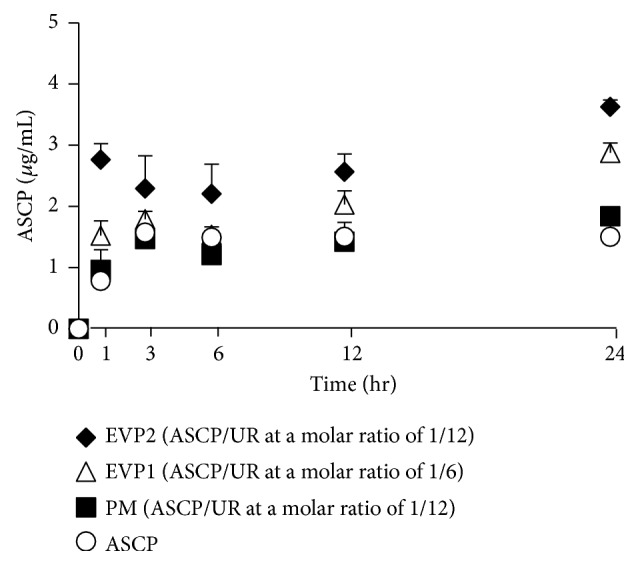
Solubility profiles of ASCP/UR systems. Results are expressed as the mean ± SD (*n* = 3).

Evaluation of its crystalline state suggested that EVP2 (ASCP/UR at a molar ratio of 1/12) forms complexes. Therefore, the solubility of ASCP as a result of complex formation was investigated (Figure 7). After shaking for 1 h, the solubility of ASCP was as follows: ASCP: 0.78 ± 0.21 *μ*g/mL, the PM (ASCP/UR at a molar ratio of 1/12): 0.96 ± 0.33 *μ*g/mL, EVP1 (ASCP/UR at a molar ratio of 1/6): 1.52 ± 0.24 *μ*g/mL, and EVP2 (ASCP/UR at a molar ratio of 1/12): 2.76 ± 0.21 *μ*g/mL. Compared to ASCP, EVP2 (ASCP/UR at a molar ratio of 1/12) had improved solubility about 3.5-fold. This increase in solubility was thought to be due to ASCP/UR complex formation. The difference in solubility between EVP1 (ASCP/UR at a molar ratio of 1/6) and EVP2 (ASCP/UR at a molar ratio of 1/12) is thought to depend on the molar ratio of complex formation. NIR results suggested that an excessive amount of ASCP is present in EVP1 (ASCP/UR at a molar ratio of 1/6). Since free ASCP exists in EVP1 (ASCP/UR at a molar ratio of 1/6), the solubility of EVP1 (ASCP/UR at a molar ratio of 1/6) is presumably lower than that of EVP2 (ASCP/UR at a molar ratio of 1/12). The solubility of EVP1 (ASCP/UR at a molar ratio of 1/6) was half that of ASCP and EVP2 (ASCP/UR at a molar ratio of 1/12). These findings suggested the presence of ASCP/UR complexes at a molar ratio of 1/1 in EVP1 (ASCP/UR at a molar ratio of 1/6).

### 3.6. DPPH Radical Scavenging Test

**Figure 8:**
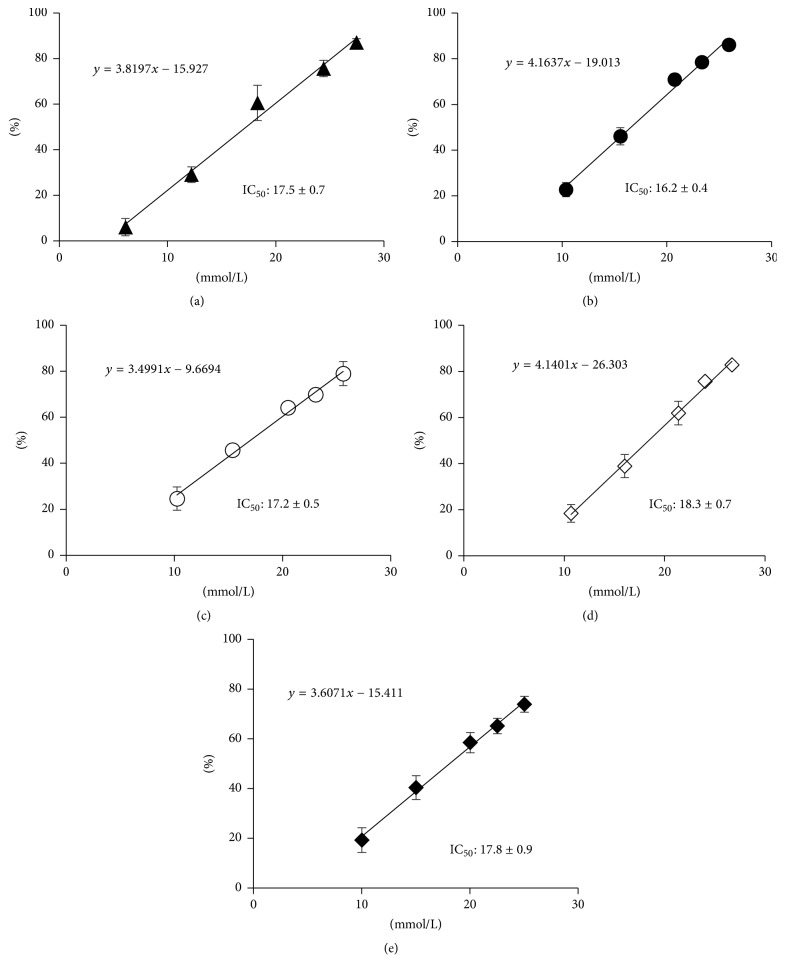
DPPH radical scavenging test of ASCP/UR systems. Results are expressed as the mean ± SD (*n* = 3). (a) AA, (b) ASCP, (c) PM (ASCP/UR at a molar ratio of 1/12), (d) EVP1 (ASCP/UR at a molar ratio of 1/6), and (e) EVP2 (ASCP/UR at a molar ratio of 1/12).

A DPPH radical scavenging test was conducted to investigate whether the antioxidant capacity of ASCP was retained along with ASCP/UR complex formation (Figure 8). In addition, the antioxidant capacity of ASCP/UR was compared to that of AA. IC_50_ for DPPH radical scavenging ability was 17.5 ± 0.7 mmol/L for AA, 16.2 ± 0.4 mmol/L for ASCP, 17.2 ± 0.5 mmol/L for the PM (ASCP/UR at a molar ratio of 1/12), 18.3 ± 0.7 mmol/L for EVP1 (ASCP/UR at a molar ratio of 1/6), and 17.8 ± 0.9 mmol/L for EVP2 (ASCP/UR at a molar ratio of 1/12). IC_50_ of EVP2 (ASCP/UR at a molar ratio of 1/12) did not differ significantly from that of ASCP. This antioxidant capacity was presumably retained because ASCP/UR complex formation does not involve the carbonyl group of the lactone ring, which contributes to the antioxidant action of ASCP.

### 3.7. Mushroom Tyrosinase Inhibition Assay

**Figure 9:**
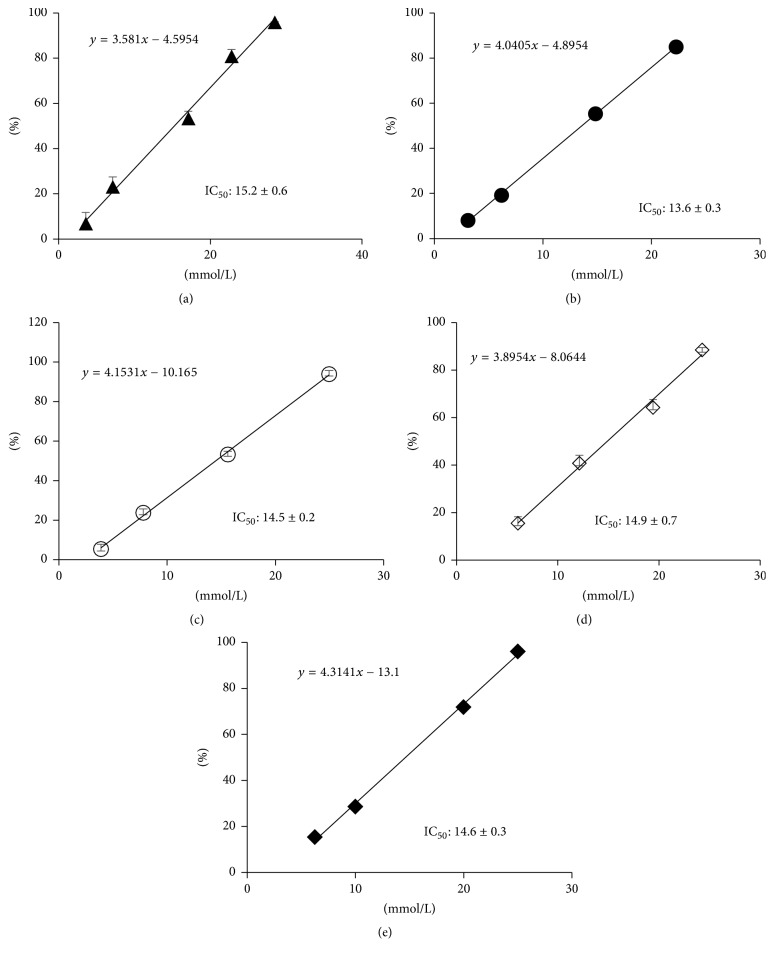
Mushroom tyrosinase inhibition assay of ASCP/UR systems. Results are expressed as the mean ± SD (*n* = 3). (a) AA, (b) ASCP, (c) PM (ASCP/UR at a molar ratio of 1/12), (d) EVP1 (ASCP/UR at a molar ratio of 1/6), and (e) EVP2 (ASCP/UR at a molar ratio of 1/12).

ASCP is reported to have whitening action by suppressing melanin production [4, 5]. To investigate whether whitening action was retained by the ASCP/UR complexes prepared in this study, a mushroom tyrosinase inhibition assay was performed (Figure 9). IC_50_ for inhibition of mushroom tyrosinase activity was compared as an indicator of the inhibition of melanin production. IC_50_ for inhibition of mushroom tyrosinase activity was 15.2 ± 0.6 mmol/L for AA, 13.6 ± 0.3 mmol/L for ASCP, 14.5 ± 0.2 mmol/L for the PM (ASCP/UR at a molar ratio of 1/12), 14.9 ± 0.7 mmol/L for EVP1 (ASCP/UR at a molar ratio of 1/6), and 14.6 ± 0.3 mmol/L for EVP2 (ASCP/UR at a molar ratio of 1/12). IC_50_ of EVP2 (ASCP/UR at a molar ratio of 1/12) did not differ significantly from that of ASCP, suggesting that action to inhibit melanin production was retained even when ASCP/UR complexes were formed. Retention of the ability to inhibit melanin production was presumably due to fact that UR interacts with the alkyl group of ASCP without involving its lactone ring, which is responsible for its inhibition of melanin production.

## 4. Conclusion

DSC, PXRD, SEM, and NIR revealed molecular interaction between ASCP and UR. Results also indicated the molar ratio of inclusion for the EVP (ASCP/UR at a molar ratio of 1/12) and they indicated that UR with a hexagonal crystal system included ASCP in its voids.

Improvement in the solubility of ASCP was noted as a result of the formation of ASCP/UR complexes. In addition, the formation of ASCP/UR complexes resulted in no change in the DPPH radical scavenging ability or the mushroom tyrosinase inhibition activity of ASCP. These findings suggest that ASCP/UR complexes are a useful means of improving solubility while retaining the action of ASCP. ASCP has antioxidant and whitening action in the epidermis, so skin permeation is required for ASCP to have an effect.

The current study attempted to form complexes with UR as a means of improving the solubility of ASCP. UR has action to promote skin penetration. Accordingly, ASCP/UR complexes may possess action to promote skin penetration. Further studies are needed in order to determine how ASCP/UR complex formation affects the skin permeation of ASCP.
